# Physiological and Horticultural Responses of HLB-Affected ‘Valencia’ Orange Trees to Trunk-Injected Oxytetracycline and Streptomycin Formulations Differing in Adjuvant and pH

**DOI:** 10.3390/antibiotics15090892

**Published:** 2026-09-11

**Authors:** Igor Silva, Jorge Pereira, Swadeshmukul Santra, Ute Albrecht

**Affiliations:** 1 Southwest Florida Research and Education Center, Horticultural Sciences Department, Institute of Food and Agricultural Sciences, University of Florida, Immokalee, FL 34142, USA; igorrodriguesdas@ufl.edu; 2 NanoScience Technology Center, University of Central Florida, Orlando, FL 32826, USA; jorge.pereira@ucf.edu (J.P.); swadeshmukul.santra@ucf.edu (S.S.); 3 Department of Chemistry, University of Central Florida, Orlando, FL 32816, USA; 4 Burnett School of Biomedical Sciences, University of Central Florida, Orlando, FL 32827, USA

**Keywords:** citrus greening, bacterial disease, antimicrobial, vascular delivery, disease management

## Abstract

Background/Objectives: Trunk injection of oxytetracycline (OTC) can suppress *Candidatus* Liberibacter asiaticus (*C*Las) and improve the productivity of citrus trees affected by huanglongbing (HLB), a devastating bacterial disease, but conventional acidic formulations may cause phytotoxicity and exacerbate trunk injury. This study evaluated a proprietary adjuvant intended to stabilize OTC at higher pH and assessed streptomycin (STM) as an alternative or complement to OTC. Methods: Two commercial field trials with HLB-affected ‘Valencia’ sweet orange trees compared a 2 × 4 factorial combination of two formulations, adjuvant/high-pH and no-adjuvant/low-pH, and four antibiotic treatments: none, OTC, STM, and OTC + STM. Tree growth, canopy health, phytotoxicity, *C*Las load, external wound responses, fruit drop, yield, and fruit quality were evaluated. Results: Tree growth and canopy health were not affected by treatment. The adjuvant/high-pH formulation caused severe canopy phytotoxicity in trial 1 and increased wound width, wound length, and bark cracking in both trials, compared to the control. OTC and OTC + STM generally reduced fruit drop, increased yield, and improved fruit quality attributes though effects on *C*Las were moderate and not consistent. STM alone provided no consistent therapeutic or horticultural benefit, and OTC + STM were not consistently superior to OTC alone. Conclusions: OTC was the primary effective component to mitigate HLB in affected citrus trees. The adjuvant/high-pH formulations did not affect OTC efficacy but caused tree injury, even in the absence of OTC. Novel trunk-injection formulations must balance chemical stability and delivery with plant tissue compatibility and safety to meet commercial viability.

## 1. Introduction

Citrus huanglongbing (HLB) remains one of the most destructive diseases affecting citrus production worldwide, severely compromising tree health, fruit quality, and long-term grove sustainability [1,2,3]. In Florida, HLB has been present since its first confirmation in 2005 and has led to significant reductions in citrus acreage and productivity, reinforcing the urgent need for effective management strategies [2,4,5]. The disease is associated with the phloem-limited bacterium *Candidatus* Liberibacter asiaticus (*C*Las), which is transmitted by the Asian citrus psyllid *Diaphorina citri*. Following infection, *C*Las colonizes the phloem and disrupts vascular transport, resulting in characteristic symptoms such as leaf chlorosis and blotchy mottle, twig dieback, premature fruit drop, and progressive tree decline [6,7,8].

Among currently available management tools, trunk injection of oxytetracycline (OTC) has emerged as an effective approach to suppress *C*Las populations and improve tree performance under HLB-endemic production conditions [9,10,11]. This technique allows the direct delivery of the antibiotic into the vascular system, facilitating systemic distribution [12,13]. Studies have demonstrated that OTC trunk injections can reduce bacterial titers, decrease fruit drop, and improve yield and fruit quality in HLB-affected citrus trees [9,10,11]. However, despite the benefits, several limitations remain to be addressed to optimize and ensure the long-term sustainability of this strategy.

First, OTC formulations registered for trunk injection require acidification to a pH of 1.8–2.0 to ensure solubility and stability of the antibiotic [14,15,16,17]. The acidic conditions may contribute to phytotoxic effects, including bark cracking, vascular tissue damage, and impaired wound healing at the injection site. Second, low-pH formulations limit the compatibility of OTC with other crop protection materials, restricting the potential for combined or integrated treatment approaches. Third, the injection process itself creates trunk wounds that must be compartmentalized by the tree to prevent decay [12,18,19,20], and repeated applications may compromise the trunk’s integrity over time.

Furthermore, as OTC injection therapy has been widely adopted in Florida [21,22], there are concerns regarding the development of antibiotic resistance [23,24]. The integration of alternative antibiotics, such as streptomycin (STM), has been proposed as a strategy to prevent or delay resistance and enhance disease management [13,23,25,26]. However, the effectiveness and behavior of STM when applied via trunk injection remain less well understood than its established use in foliar applications [13,26].

To address these limitations, recent efforts have focused on developing adjuvants designed to stabilize and maintain or enhance OTC efficacy at a higher pH (7.0–8.0), potentially minimizing injury at the injection site and enabling the co-application of additional compounds. This study evaluated the physiological and horticultural responses of citrus trees injected with OTC and STM using formulations with and without a novel adjuvant and differing in pH. The higher-pH OTC formulation containing the adjuvant was hypothesized to provide an effective alternative to conventional low-pH formulations and allow compatibility with other crop protection products. STM was included to assess its potential as an alternative or complement to OTC.

## 2. Results

### 2.1. Tree Size and Canopy Health

Treatment effects on tree growth were evaluated based on the growth increment from 0 to 12 months after injection. No significant effects of adjuvant, antibiotic treatment, or their interaction were detected for rootstock diameter, scion diameter, or canopy volume increment in trial 1 (Table S1). Likewise, no treatment effects were observed for tree growth in trial 2 (Table S2).

Canopy health was evaluated based on HLB severity and canopy density ratings at the end of each season. No significant effects of adjuvant treatment, antibiotic treatment, or their interaction were detected in trial 1 (Table S3) or trial 2 (Table S4).

### 2.2. Phytotoxicity

**Figure 1:**
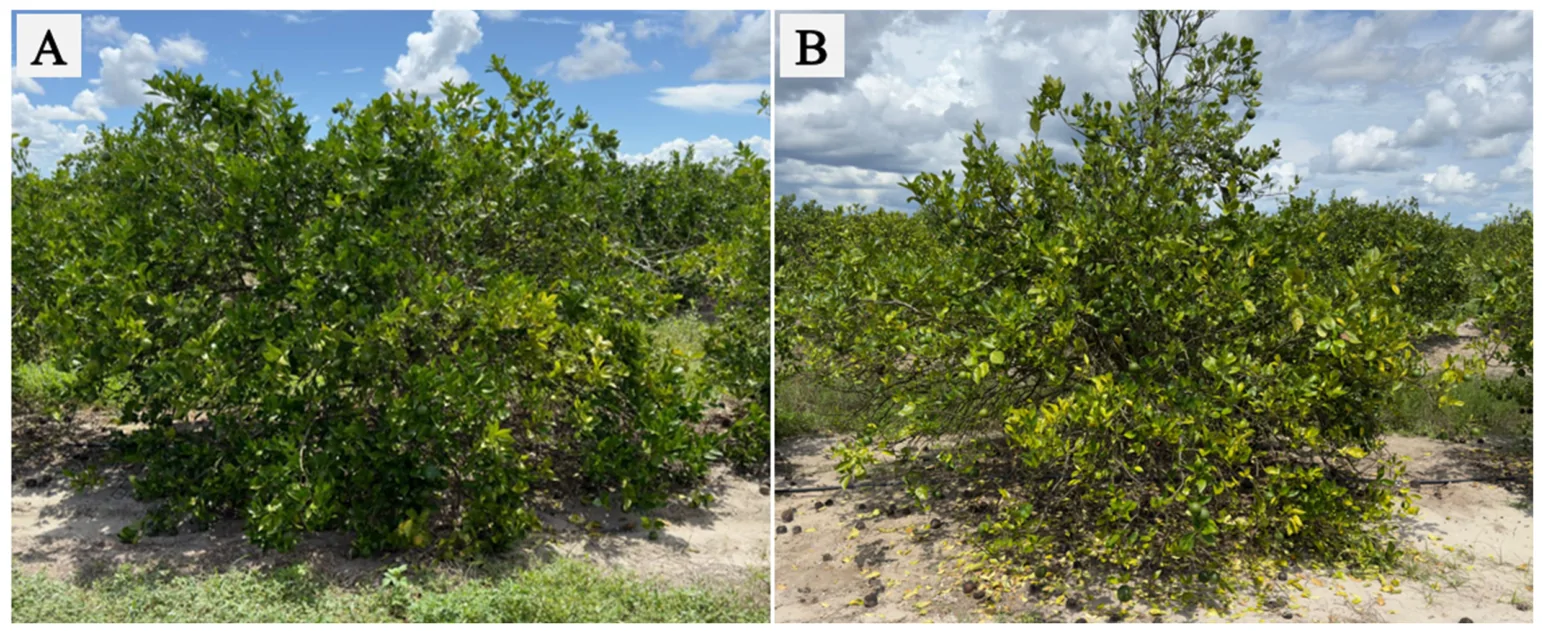
Visual comparison of canopy phytotoxicity and leaf drop symptoms in ‘Valencia’ orange trees 12 days after injection in trial 1. (**A**) Tree injected with the no-adjuvant/low-pH formulation without antibiotic (pH 1.9). (**B**) Tree injected with the adjuvant/high-pH formulation without antibiotic (pH 8.1).

Severe phytotoxicity symptoms, including leaf yellowing and bronzing (canopy phytotoxicity) and leaf drop, were observed in trial 1 following injection, whereas no apparent treatment-related phytotoxicity symptoms were observed in trial 2. Therefore, phytotoxicity was quantified only in trial 1. In that trial, both canopy phytotoxicity and leaf drop were significantly affected by adjuvant treatment (Table 1). Trees injected with the adjuvant (high pH) had higher leaf drop and canopy phytotoxicity ratings (3.5) than trees injected without adjuvant (low pH), which had ratings of 1.6 and 1.5, respectively (Figure 1). Antibiotic treatment also significantly affected both canopy phytotoxicity and leaf drop. The highest ratings were observed for trees that received OTC + STM (2.8) and the lowest for control and STM-injected trees (2.4 and 2.3, respectively). There were significant interactions for both variables, and ratings were significantly higher for the adjuvant without antibiotic (3.7 for both variables) than the no-adjuvant control (1.1 for both variables) (Table S5).

### 2.3. Candidatus Liberibacter asiaticus Detection

*Candidatus* Liberibacter asiaticus was assessed by real-time polymerase chain reaction and expressed as Ct-values, with higher Ct-values indicating a lower bacterial load and vice versa. Ct-values fluctuated over time in both trials (Table 2). No significant effects of adjuvant treatment were detected at any sampling time in either trial, but antibiotic treatment affected Ct-values at specific evaluation times.

In trial 1, three months after injection (MAI), trees treated with OTC and OTC + STM had significantly higher Ct-values (28.8 and 28.9, respectively) than trees treated with STM (26.7). No significant antibiotic effects were detected at 6 or 9 MAI. A significant interaction between adjuvant and antibiotic was detected at 9 MAI, but mean separation did not indicate differences among combinations (Table S6). Repeated measures ANOVA indicated that in addition to antibiotic treatment, time was a significant factor affecting Ct-values (Table S7).

In trial 2, antibiotic treatment significantly affected Ct-values at 3 and 6 MAI. At 3 MAI, trees treated with OTC had a significantly higher Ct-value (26.2) than trees treated with STM (24.9), whereas the control and OTC + STM treatments did not differ significantly from either treatment. At 6 MAI, trees treated with OTC and OTC + STM had significantly higher Ct-values (25.8 and 25.5, respectively) than trees treated with STM (23.9), while the control did not differ significantly from any antibiotic treatment. No significant interaction between the adjuvant and the antibiotic was detected. Repeated measures ANOVA indicated that in addition to antibiotic treatment, time was a significant factor affecting Ct-values (Table S7).

### 2.4. Wound Responses to Injection

In trial 1, trees treated with the adjuvant (high pH) consistently showed significantly greater wound injury than trees without the adjuvant (low pH) (Table 3). Three months after injection, trees treated with the adjuvant had higher mean injection hole scores (2.7), indicating less hole closure, than trees without adjuvant (2.4), while wound widths were 1.4 cm and 1.0 cm, respectively; wound lengths were 6.1 cm and 1.7 cm, respectively, and bark crack incidence was 83% and 28%, respectively. Antibiotic treatment was only significant for injection hole score at this time point, with OTC and OTC + STM producing the highest ratings (2.8) and the control the lowest (2.2). However, a significant adjuvant × antibiotic interaction was detected for injection hole score and wound width. For injection hole score, control trees treated with adjuvant alone and trees treated with OTC + STM without adjuvant had significantly higher ratings (2.6 and 2.7, respectively), indicating less hole closure, than control trees without adjuvant (1.7) (Table S8). For the wound width, except for OTC + STM, all treatments containing the adjuvant caused significantly wider wounds (1.9 cm) than control trees, OTC, and STM-treated trees without adjuvant (1.0 cm) (Table S8).

**Figure 2:**
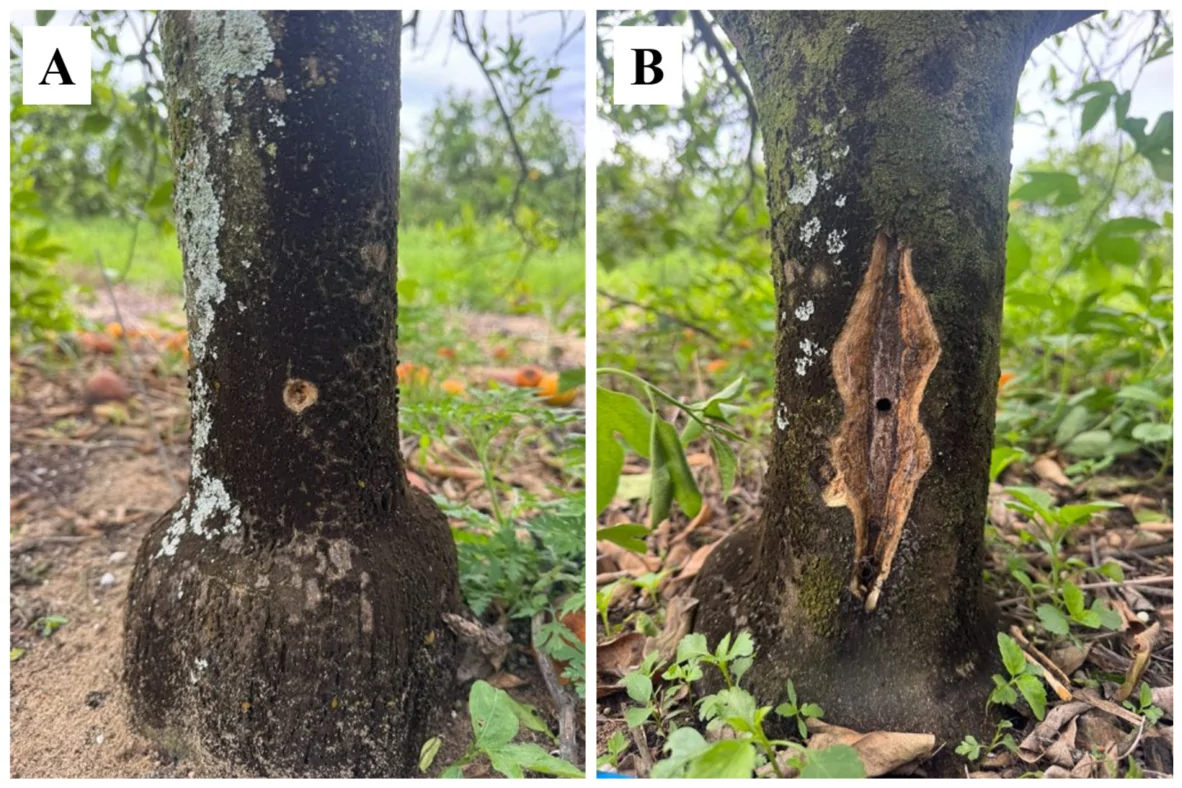
Visual comparison of trunk wound phenotypes of ‘Valencia’ orange trees nine months after injection in trial 2. (**A**) Tree treated with the no-adjuvant/low-pH formulation without antibiotic (pH = 1.9). (**B**) Tree treated with the adjuvant/high-pH formulation containing OTC + STM (pH = 7.8). Photos are approximately to scale.

Nine months after injection in trial 1, trees treated with adjuvant continued to have wider and longer wounds, and a higher bark crack incidence than trees without adjuvant (Table 3 and Figure 2). The mean wound width was 2.5 cm in trees treated with adjuvant and 1.5 cm in trees without adjuvant, while the mean wound length was 6.8 and 2.1 cm, respectively. Bark crack incidence was 70% and 27%, respectively. Injection hole score was not significantly affected by adjuvant treatment at this evaluation time. Antibiotic treatment significantly affected injection hole score, wound length, and crack incidence. Trees injected with OTC or OTC + STM had higher injection hole scores (2.2 and 2.1, respectively), indicating less hole closure, than trees in the control and STM treatments (1.5 and 1.7, respectively). Although antibiotic treatment affected wound length significantly at 9 months, mean separation was not significant. Bark crack incidence was significantly higher in trees injected with OTC + STM (63%) than in control trees (31%). There were no significant interactions at this time point.

Repeated measures ANOVA indicated that in addition to antibiotic treatment, time was a significant factor affecting all measured wound variables (Table S9), with injection hole scores becoming lower (indicating more wound closure) and wound width and length becoming larger with increasing time after injection. Interactions were significant for adjuvant × antibiotic (injection hole score), adjuvant × time (injection hole score and wound width), and antibiotic × time (wound length).

In trial 2, a similar pattern was observed as in trial 1. Three months after injection, trees treated with adjuvant had a slightly higher overall mean injection hole score (3.00), indicating less hole closure, than trees without adjuvant (2.96). A significant adjuvant × antibiotic interaction was detected for injection hole score; however, mean separation showed no significant differences among the individual treatment combinations (Table S8). Adjuvant treatment also significantly affected wound width, wound length, and bark crack incidence. Trees treated with adjuvant had wider and longer wounds (1.3 cm and 7.4 cm, respectively) and a higher bark crack incidence (88%) compared with trees without adjuvant (1.0 cm, 1.8 cm, and 32%, respectively) (Table 3). Antibiotic treatment only affected the wound length significantly. Trees treated with OTC + STM had longer wounds (6.6 cm) than control trees and trees injected with STM (3.5 cm).

Nine months after injection, adjuvant treatment significantly affected all wound variables in trial 2 (Table 3 and Figure 2). Trees treated with adjuvant had higher injection hole scores (2.6), wider (2.2 cm) and longer (8.5 cm) wounds, and a higher bark crack incidence (96%) than trees without adjuvant (2.0, 1.1 cm, 2.3 cm, and 40%, respectively). Antibiotic treatment also affected all wound variables significantly. Trees injected with OTC had a higher injection hole score (2.6), indicating less closure, than trees injected with STM (2.0). Wounds were wider in trees injected with OTC + STM (2.0 cm) than in control trees and trees injected with STM (1.4 cm). Wounds were longest in trees injected with OTC + STM (8.2 cm), followed by trees injected with OTC (5.8 cm), while control trees and trees injected with STM had the shortest wounds (3.6 cm and 4.1 cm, respectively). Bark crack incidence was higher in trees injected with OTC + STM (91%) than in control trees and trees injected with STM (50% and 54%, respectively). A significant adjuvant × antibiotic interaction was detected for bark crack incidence, but mean separation showed no significant differences among the treatment combinations (Table S8).

Repeated measures ANOVA indicated that in addition to antibiotic treatment, time was a significant factor affecting all measured wound variables except bark crack incidence (Table S9), with injection hole scores becoming lower (indicating more wound closure) and wound width and length becoming larger with increasing time after injection. Interactions were significant for adjuvant × antibiotic (injection hole score), adjuvant × time (injection hole score, wound width, and wound length), antibiotic × time (injection hole score, wound width, and wound length), and adjuvant × antibiotic × time (injection hole score and wound width).

### 2.5. Fruit Drop, Yield, and Fruit Quality

In trial 1, adjuvant treatment did not significantly affect fruit drop, yield, and fruit quality. In contrast, antibiotic treatment affected all measured variables significantly (Table 4). Control trees had the most fruit drop (7.4%), whereas trees injected with OTC + STM had the least (5.4%). Yield was lower in control trees (21.2 kg tree^−1^) than in trees injected with OTC + STM (30.5 kg tree^−1^) or OTC alone (28.9 kg tree^−1^). Trees injected with OTC + STM and OTC alone produced heavier (135 g and 140 g, respectively) and larger fruits (152 cm^3^ and 153 cm^3^, respectively) than control and STM-injected trees, which had fruit weights of 119 g and 120 g and fruit sizes of 124 cm^3^ and 127 cm^3^, respectively. Peel color values were higher in trees injected with OTC + STM and OTC alone (−4.7 and −4.8, respectively) than in control and STM-injected trees (−7.1 and −6.8, respectively). No significant adjuvant × antibiotic interactions were detected.

In trial 2, adjuvant treatment also had no significant effect on fruit drop, yield, and fruit quality (Table 4). Antibiotic treatment significantly affected fruit drop and yield. Control trees had the most fruit drop (17.3%), whereas trees injected with OTC had the least (12.9%). Yield was higher in trees injected with OTC (21.3 kg tree^−1^) than in control trees and trees injected with STM (16.5 kg tree^−1^ and 17.2 kg tree^−1^, respectively). The adjuvant × antibiotic interaction was significant only for fruit size, but mean separation did not detect significant differences among treatment combinations (Table S10).

### 2.6. Juice Quality

In trial 1, adjuvant treatment did not significantly affect any of the measured juice quality variables. In contrast, antibiotic treatment significantly affected TSS, TSS/TA, pounds solids, and juice color (Table 5). OTC + STM and OTC treatments produced higher TSS values (9.8 °Brix), higher TSS/TA ratios (17.1 and 16.9, respectively), and more pounds solids per box (5.1 lbs.) than the control and STM treatments, which produced values of 8.9 °Brix (TSS), 14.6 (TSS/TA), and 4.6 and 4.7 lbs. solids per box, respectively. The juice color was highest in OTC-treated trees (36.5) and lowest in control trees (36.1).

In trial 2, adjuvant treatment significantly affected TSS, TA, TSS/TA, and pounds solids per box (Table 5). Trees treated without adjuvant produced higher TSS and TA values (9.9 °Brix and 1.03%, respectively), a lower TSS/TA ratio (9.7), and more pounds solids per box (4.8 lbs.) than trees treated with adjuvant (9.7 °Brix, 0.98%, 10.0, and 4.6 lbs., respectively). Antibiotic treatment significantly affected all measured juice quality variables. Trees injected with OTC and OTC + STM produced higher TSS values (10.2 and 10.1 °Brix, respectively) than control and STM-injected trees (9.4 and 9.3 °Brix, respectively). Control trees produced a significantly higher TA (1.04%) than OTC + STM-injected trees (0.96%). Trees injected with OTC and OTC + STM also produced higher TSS/TA ratios (10.4 and 10.6, respectively), more pounds solids per box (4.9 and 4.8 lbs., respectively), and a better juice color (36.0 and 35.8, respectively) than control trees and trees injected with STM, which produced TSS/TA ratios of 9.1 and 9.3, respectively, 4.4 lbs. solids per box, and juice color values of 35.3 and 35.4, respectively. There were no significant adjuvant × antibiotic interactions.

## 3. Discussion

Tree growth was not affected by adjuvant, antibiotic treatment, or their interaction during the course of the trials. The lack of treatment effects on tree size indicates that the improvements observed in yield and fruit quality were not associated with detectable changes in structural growth within the first 12 months after injection. This is not surprising because changes in trunk diameter or canopy volume may require more than one growing season to become measurable, especially in HLB-affected trees, where growth is already limited by impaired phloem function, carbohydrate imbalance, root loss, and canopy decline [27,28,29]. Similarly, other OTC trunk injection studies have reported improvements in yield and fruit quality, although tree size responses were variable, with Archer et al. [30] reporting increases in rootstock and scion circumference, and Albrecht et al. [31] reporting no significant effects on canopy volume, scion diameter, or rootstock diameter. Therefore, yield and fruit quality appeared to be more sensitive indicators of treatment performance than biometric variables during the evaluation period.

Severe canopy phytotoxicity was observed only in trial 1 when trees were injected with the adjuvant (high pH), regardless of the antibiotic. This suggests that the phytotoxic response was mainly associated with the adjuvant rather than the antibiotic alone. However, no negative effects on canopy density or yield were detected at the end of the season, suggesting that the early phytotoxicity did not result in persistent effects on these variables. Phytotoxic effects have been a historical concern with antibiotic trunk injection in citrus, and earlier tetracycline injection studies were limited in part by concerns about tree injury following injection [32,33]. More recent work also indicates that phytotoxicity and wound compartmentalization can be affected by the injected formulation, including the active ingredient, pH, and other formulation components [18,31]. The absence of phytotoxicity in trial 2 suggests that the reformulated adjuvant had reduced phytotoxicity, though environmental factors and the tree physiological status at the time of injection may contribute to whether phytotoxic effects occur or not. Therefore, crop protection formulations administered by trunk injection need to be evaluated not only for chemical stability and efficacy, but also for compatibility with living citrus tissues under different environmental conditions.

Increases in Ct-values, indicative of a reduced *C*Las load, were noted at specific time points after injection. This response was driven mainly by antibiotic treatment but not by the adjuvant, with OTC and OTC + STM generally resulting in higher Ct-values. These results suggest that OTC can temporarily inactivate the bacteria, although responses were moderate and varied across sampling times. Previous studies also reported OTC effects on *C*Las in citrus leaves and roots following trunk injection, although the timing and duration of the response varied among studies [9,10,18,34]. The inconsistent response over time may reflect the complex biology of *C*Las distribution and antibiotic activity in HLB-affected trees. *C*Las is unevenly distributed within citrus canopies and roots, and bacterial titers fluctuate with tissue age, season, tree condition, and vector-mediated reinfection [28,35,36]. In addition, OTC is generally considered bacteriostatic rather than bactericidal, meaning that it may suppress bacterial multiplication without eliminating bacterial DNA from infected tissue. Previous studies have suggested that *C*Las DNA may remain detectable after bacterial cells are no longer active, which may delay measurable changes following antibiotic treatment [37].

Streptomycin (STM) alone did not provide a therapeutic response in any of the trials. Ct-values were similar to or lower than those of control trees, and fruit drop, yield, or fruit quality were not improved. These results suggest that, despite its demonstrated antibacterial activity against Liberibacter species [38,39,40,41], STM may not have reached or maintained effective concentrations in the tissues where *C*Las is located. This interpretation is supported by other studies comparing the uptake and distribution of OTC and STM in citrus. Specifically, Al-Rimawi et al. [25] showed that both antibiotics can be taken up and translocated in young citrus seedlings, but only OTC was detected at high levels in leaves, xylem, and phloem. In contrast, STM accumulated more strongly in the roots, suggesting a greater association with the xylem. Vincent et al. [13] also reported that STM was poorly delivered systemically compared with OTC and suggested that low leaf concentrations or binding to xylem-associated tissues may limit its efficacy against *C*Las. Thus, the lack of response to STM in the present study is likely related to the lack of translocation to the phloem. However, larger doses and different pH conditions should be evaluated before dismissing STM injections as a treatment option for HLB management.

Early plant antibiotic studies also support the idea that uptake and translocation of STM can occur, but are highly dependent on plant species, delivery route, concentration, and tissue interaction. Specifically, streptomycin was shown to move into the foliage of peach seedlings after root uptake, but its distribution differed among leaves, and higher concentrations caused reduced apical growth, chlorosis, and necrotic symptoms [42]. Other early studies demonstrated that streptomycin and other antibiotics can be absorbed and redistributed within plants following root uptake, exposure of cut shoots or leaves, and seed treatment, although accumulation, persistence, and phytotoxicity varied among compounds and plant species [43,44,45]. This shows that systemic movement alone does not guarantee disease control. For an antibiotic to suppress an internal pathogen, it must be active against the pathogen, absorbed by the host, transported to the correct tissue, remain stable after uptake, and be present at biologically effective concentrations [44]. The present results suggest that STM may have entered the tree but did not produce a reduction in *C*Las or improvement in horticultural performance.

The combination of OTC + STM improved several of the measured plant physiological variables, especially in trial 1, but was generally not superior to OTC alone. In both trials, OTC and OTC + STM resulted in less fruit drop, higher yield, and improved fruit and juice quality compared with the control and STM treatments. This suggests that the benefits of the combined treatment were driven primarily by OTC. Previous work in citrus reported strong *C*Las suppression after trunk injection of different antibiotic combinations and suggested that combining a bacteriostatic antibiotic, such as OTC, with a bactericidal antibiotic, such as STM, could improve disease control [34]. In that study, the combination of OTC and STM showed the strongest suppression of *C*Las as well as significant yield increases compared with water injection but yield effects were not compared against each antibiotic alone. The fruit yield and quality responses observed in the present study are also consistent with previous studies in which OTC trunk injection reduced fruit drop, increased yield, increased fruit size, and improved juice quality in HLB-affected citrus trees [9,11,46]. Notably, cumulative benefits were found after two consecutive years of OTC injection, with economic benefits outweighing treatment costs depending on the application strategy [9]. Antibiotic combination and adjuvant strategies may improve antimicrobial performance in some systems, but their success depends on compound compatibility, delivery, persistence, and target-site exposure [47,48,49].

The improvement in fruit and juice quality in response to OTC injection may reflect partial recovery of physiological functioning in HLB-affected trees. HLB disrupts phloem transport, carbohydrate partitioning, source–sink relationships, water relations, and fruit maturation, resulting in small, poorly colored fruits with lower juice quality and increased pre-harvest fruit drop [27,29,50,51,52]. Citrus fruit growth and maturation depend on sustained carbohydrate supply, water movement, hormonal regulation, and vascular function [53]. Therefore, reductions in bacterial pressure after OTC injection may allow better carbohydrate accumulation and fruit development, which could explain the higher TSS, TSS/TA ratio, fruit weight, fruit size, juice color, and peel color observed in this and previous studies [11]. Moreover, Castellano-Hinojosa et al. [54] reported positive correlations between the relative abundance of some bacterial groups in the bark, rhizosphere, and fruit microbiomes and fruit yield and weight, suggesting possible indirect microbiome-related effects.

OTC was effective with and without the adjuvant, and the adjuvant generally did not affect OTC effects on fruit yield and fruit quality variables, except in trial 2, where it produced lower TSS, TA, and pounds solids per box values than no-adjuvant treatment, although differences were moderate. Differences in the adjuvant as well as in location, tree age, previous injection history, and weather events may have contributed to the different juice quality responses between trials. Adjuvants are commonly used to improve agrochemical performance by modifying solubility, stability, retention, penetration, spreading, compatibility, or uptake, but their effects are highly dependent on the active ingredient, formulation, application method, target tissue, and environmental context [55,56]. Importantly, adjuvants are not biologically inert; they can alter plant tissue interaction and may have their own phytotoxic or physiological effects [56]. The present results support this concept because the adjuvant used in our study exerted phytotoxic effects in trial 1 and exacerbated trunk injury without improving antibiotic efficacy.

The rationale for evaluating an alternative OTC formulation remains scientifically relevant. Commercial OTC formulations require acidification for dissolution, and highly acidic formulations raise concerns about tissue compatibility, phytotoxicity, and trunk injury. However, pH cannot be evaluated independently because the high-pH solutions require additional chemicals (adjuvants), whereas the low-pH solutions do not. Therefore, the effects on phytotoxicity and/or trunk injury observed with the adjuvant/high-pH formulation cannot be attributed specifically to the pH. They may instead reflect effects of adjuvant components, differences in viscosity, chemical interactions among formulation components, or a combination of these factors. However, within the conditions tested, the adjuvant-containing high-pH OTC formulation performed similarly to the low-pH formulation without adjuvant, demonstrating that OTC can be injected at higher pH without affecting its performance.

Earlier work on tetracycline trunk injection in HLB-affected citrus trees found no significant differences in efficacy among solutions ranging from pH 3 to 8, although phytotoxicity was observed at all tested pH values, except pH 4, and wood discoloration was observed in most injected trees [33]. Studies on tetracycline formulation chemistry indicate that OTC is challenging to formulate because of solubility limitations, chemical instability, and interactions with complexing agents or ions [57]. Studies conducted in environmental matrices showed that OTC speciation, sorption, and potential bioavailability are influenced by pH, ionic strength, and complexation reactions [58,59]. Although these findings cannot be directly extrapolated to citrus tissues or used to explain the treatment effects observed here, they illustrate the sensitivity of OTC physicochemical behavior to solution conditions. Therefore, adjuvants, like the one in this study, have the potential to help formulate OTC to aid its compatibility with other agrochemicals, such as fertilizers, despite OTC’s physicochemical behavior in solution.

In addition to the chemical characteristics of the injected compound, uptake and distribution of trunk-injected compounds are affected by tree species, tissue anatomy, season, transpiration, tree health, injection system, injector size, and other factors [60,61,62,63], which all contribute to their biological efficacy. VanWoerkom et al. [63] reported that inconsistent delivery of some trunk-injected compounds in apple trees may have been related to molecular or chemical properties, including compound size, viscosity, pH, and compatibility with vascular movement. In the present study, the dissolution of OTC with the high pH adjuvant did not significantly affect *C*Las suppression, yield, or fruit quality traits. However, uptake time was not quantified, and OTC stability, movement, and distribution within the tree were not directly assessed, therefore not allowing any conclusions regarding adjuvant effects on OTC stability or distribution.

The external wound response was the clearest negative effect associated with the adjuvant/high-pH formulation. Across both trials, trees injected with adjuvant had wider and considerably longer wounds and a higher bark crack incidence than trees injected without adjuvant. Importantly, considerable trunk injury in response to the adjuvant was noted in trial 2, even though no canopy phytotoxicity was observed. This suggests that phytotoxicity and localized trunk injury may be associated with different components in the formulation and should be evaluated separately when testing new injectable formulations [18,19].

The increased wound injury observed in this study is consistent with previous reports showing that trunk injection can cause external and internal damage, and that the extent of injury depends on the injected compound, formulation, injection method, injection site, season, and tree condition [18,19,64,65]. The CODIT (Compartmentalization of Decay in Trees) model describes how trees compartmentalize wounds to limit the spread of decay, but successful compartmentalization also involves the formation of chemically and anatomically altered tissues around the wound [20,66]. In citrus, Archer and Albrecht [18] showed that trees effectively compartmentalized wounds caused by water injection, but OTC injection impeded wound healing and increased internal damage compared with water injection. The present study extends this concept by showing that formulation components can strongly affect external wound severity, regardless of the pH of the injected solution.

The results from this study also reinforce the need to interpret trunk injection as a benefit–damage tradeoff. Smith [67] emphasized that trunk injection requires wounding the tree to deliver therapeutic compounds, and the value of the treatment depends on whether the expected benefit outweighs the biological cost of injury. Doccola et al. [64] reported that healthy green ash trees were able to compartmentalize injection wounds without signs of infection, decay, or structural damage, and that wound closure was related to tree vigor. However, other studies have shown that injection port size, drill or needle system, wound plugs, season, and formulation can alter healing and bark cracking responses [18,19]. In the present study, treatments containing OTC caused trunk injury, but they also improved yield and fruit quality. In contrast, the adjuvant/high-pH formulation increased trunk injury without enhancing tree responses. While it appears that the therapeutic benefits of OTC outweigh the negative effects, the lack of benefit of the adjuvant on tree performance is difficult to justify for injection of OTC alone and suggests other active ingredients might be needed to increase performance.

In conclusion, this proof-of-concept study shows the physiological and horticultural response of citrus trees injected with OTC and STM using formulations with and without a novel adjuvant and differing in pH. Within the conditions tested, the adjuvant-containing high-pH OTC formulation performed similarly to the low-pH formulation without adjuvant, demonstrating that OTC can be injected at neutral to mildly alkaline pH without diminishing its performance. Future studies will develop the adjuvant to further reduce trunk damage and evaluate the performance of OTC injected with micronutrients and other active ingredients.

## 4. Materials and Methods

### 4.1. Plant Material and Experimental Approach

Two experiments were conducted—both under commercial production conditions.

#### 4.1.1. Trial 1

Trees used for this experiment were seven-year-old ‘Valencia’ sweet orange (*Citrus sinensis*) trees grafted on X-639 (*Citrus reticulata* ‘Cleopatra’ × *Poncirus trifoliata*) rootstock located at a commercial farm in southwest Florida (26.332750 N, 81.275778 W). Trees had not been injected previously. At the start of the study, the average tree height was 224 cm (ranging from 174 cm to 350 cm); the average rootstock trunk diameter was 6.7 cm (ranging from 4.8 cm to 8.5 cm), and the average scion trunk diameter was 5.4 cm (ranging from 3.5 cm to 6.8 cm). HLB is endemic in Florida [4]; therefore, all trees were visibly affected by the disease at the onset of the study.

The experiment was conducted as a randomized complete block design with 8 treatments and 8 replications (4 trees per replicate), totaling 256 trees. The trial was established in June 2024 and was proposed to be conducted for up to two years, encompassing two production seasons. However, after the harvest in year 1, all trees were accidentally injected with OTC by the grower collaborator. Therefore, a second experiment was conducted at a different location but including the same treatments.

#### 4.1.2. Trial 2

Trees used for this experiment were ten-year-old ‘Valencia’ sweet orange (*C. sinensis*) grafted on Swingle (*Citrus* × *paradisi* × *P. trifoliata*) rootstock located in central Florida (27.925111 N, 81.474889 W). All trees had been injected with OTC by the grower collaborator during the previous two years (one injection per year) according to label recommendations. At the start of the study, the average tree height was 226 cm (ranging from 162 cm to 333 cm); the average rootstock trunk diameter was 17.1 cm (ranging from 11.1 cm to 22.0 cm), and the average scion trunk diameter was 10.5 cm (ranging from 7.6 cm to 15.6 cm).

The experiment was conducted as a randomized complete block design with 8 treatment combinations and 9 replications (2 trees per replicate), totaling 144 trees. The trial was established and treatments were applied in June 2025.

### 4.2. Treatments

There were eight treatment combinations in each trial, arranged as a 2 × 4 factorial, with adjuvant treatment as the first factor and antibiotic treatment as the second factor. The adjuvant factor included two levels: (i) with adjuvant (high-pH formulation) and (ii) without adjuvant (low-pH formulation). The antibiotic factor included four levels: (i) no antibiotic, (ii) OTC, (iii) STM, and (iv) OTC + STM.

Injections were performed into the scion portion of the trunk, 5 cm above the graft union, using FlexInject^TM^ injectors (TJ BioTech LLC, Buffalo, SD, USA). The injection volume was 100 mL for all trees. Each antibiotic was applied at a fixed dose of 0.825 g (a.i.) per tree, regardless of variations in tree size. The fixed per-tree dose was selected based on the average trunk diameter of the experimental trees in accordance with the label recommendations. The OTC + STM treatment contained 0.825 g (a.i.) of OTC + 0.825 g (a.i.) of STM, for a total antibiotic mass of 1.65 g (a.i.) per tree. The OTC formulation used was Rectify (95% oxytetracycline hydrochloride, Agrosource Inc., Tequesta, FL, USA). The STM formulation was an experimental material containing 88.6% streptomycin sulfate (ASI-7418, Agrosource Inc.) and is currently not registered for use in citrus. The adjuvant was a proprietary formulation provided by the University of Central Florida (patent pending 19/265,390) and is currently not commercially available. The adjuvant composition differed between trials due to a reformulation (proprietary) following severe phytotoxicity observed in trial 1. For the no-adjuvant (low-pH) treatments, the water was acidified to a pH of 1.9 using muriatic acid before adding the antibiotics. The pHs of the final solutions were 1.9 (OTC), 2.0 (STM), and 2.1 (OTC + STM).

In trial 1, the adjuvant solution without antibiotic had a pH of 8.1, while the final solution containing adjuvant and antibiotic had pH values of 7.5 (OTC), 7.9 (STM), and 7.3 (OTC + STM). In trial 2, the adjuvant solution without antibiotic had a pH of 8.5, while the final solutions containing adjuvant and antibiotic had pH values of 8.1 (OTC), 8.5 (STM), and 7.8 (OTC + STM).

All solutions were prepared in reverse osmosis (RO) water. All ingredients were fully dissolved, and all solutions were clear before injecting. Uptake of the solutions was noted to be slower for the treatments containing the adjuvant.

### 4.3. Tree Metric Assessments

Tree size and canopy health were evaluated before any treatment was applied (May 2024) and at the end of each season (June 2025 and March 2026) through biometric measurements and visual ratings to assess differences in tree size and growth from the start to the end of the study. Biometric data included scion trunk and rootstock trunk diameter (5 cm above and 5 cm below the graft union), tree height, and canopy width. Trunk diameters and canopy width were measured in two perpendicular directions and averaged. Canopy volume was calculated as (W^2^ × H)/4, where W is the average canopy width measured in two perpendicular directions and H is tree height [31]. Visual ratings of canopy density and HLB severity were conducted as described by Archer et al. [11]. Measurements were conducted for each tree, and means for each replicate were used for statistical analysis.

### 4.4. Phytotoxicity

OTC can cause phytotoxicity in the canopy in the weeks following injection, manifested as a mild yellowing or bronzing of the leaves, or in rare cases, leaf drop and twig dieback [68]. Therefore, a visual rating scale was established. Canopy phytotoxicity was rated on a scale from 1 to 4 based on the number of affected branches: 1 = some leaves with apparent phytotoxicity symptoms; 2 = 1–2 branches with apparent phytotoxicity symptoms; 3 = 3–4 branches with apparent phytotoxicity symptoms; and 4 = >4 branches with apparent phytotoxicity symptoms. Leaf drop was rated separately on a scale from 1 to 4 as follows: 1 = zero or non-apparent; 2 = few leaves (<10%); 3 = moderate defoliation (10–30%); and 4 = severe defoliation (>30%). Phytotoxicity symptoms became apparent 12 days after injection in trial 1; therefore, canopy phytotoxicity and leaf drop ratings were recorded at that time. Canopy phytotoxicity was visually assessed in both trials; however, because no apparent treatment-related symptoms were observed in trial 2, numerical ratings were recorded only for trial 1. Measurements were conducted for each tree, and means for each replicate were used for statistical analysis.

### 4.5. Candidatus Liberibacter asiaticus Detection

*Candidatus* Liberibacter asiaticus (*C*Las) was monitored in leaf tissue using TaqMan real-time polymerase chain reaction (PCR). Samples were collected prior to injection and at 3, 6, and 9 months after injection. Each sample included a composite of twelve fully expanded, mature leaves collected from the canopy on the side of the injection. For trial 1, three leaves were collected from each of the four trees in each replicate and pooled (12 leaves per replicate). For trial 2, six leaves were collected from each of the two trees in each replicate and pooled (12 leaves per replicate). Leaves were stored at −20 °C until processing. Leaves (full blades) were ground in liquid nitrogen using a mortar and pestle and used for DNA extraction using the DNeasy Plant Pro Kit (Qiagen, Valencia, CA, USA). PCR assays were conducted as previously reported [9,11] and following the protocol of Li et al. [69] using primers HLBas/HLBr and probe HLBp for detection, and COXf/COXr with COXp for normalization. Amplifications were performed over 40 cycles in a 20 µL reaction volume containing 2 µL (20–30 ng) of extracted DNA, using an Applied Biosystems QuantStudio 3 PCR system (Thermo Fisher Scientific, Waltham, MA, USA) and the iTaq Universal Probes Supermix (Bio-Rad Laboratories, Hercules, CA, USA) according to the instructions of the manufacturers. For normalization, the mean COX Ct-value was calculated across all samples, and an individual COX correction factor was obtained by dividing the COX Ct-value of each sample by the mean COX Ct-value. The *C*Las Ct-value of each sample was then divided by this correction factor to obtain the normalized Ct-value used for statistical analysis.

### 4.6. Trunk Injury

External wound measurements were conducted 3 and 9 months after injection, following the protocol from Aćimović et al. [19] with minor modifications. Wound width and length/bark cracking were measured using a measuring tape and expressed in cm. Injection hole score was visually rated on a scale of 1 to 3, with 1 = hole fully closed, 2 = hole partially closed, and 3 = hole fully open. Thus, lower scores indicated more closure, whereas higher scores indicated less closure. Bark crack incidence was assessed as “present” or “not present”. Measurements were conducted for each tree, and means for each replicate were used for statistical analysis.

### 4.7. Fruit Yield, Fruit Quality, and Juice Quality

For trial 1, fruits were harvested on 12 March 2025 to determine yield per tree and estimate pre-harvest fruit drop. Yield was calculated as total fruit weight per tree (kg tree^−1^) by harvesting all fruits. Pre-harvest fruit drop was determined by counting fallen fruits one week prior to harvest and expressing drop in percent relative to the number of retained plus dropped fruits. The number of fruits on the tree was estimated based on the average weight per fruit in each replicate. In trial 2, freezing temperatures in January 2026 resulted in fruit loss and risk for further loss before harvest. Therefore, rather than waiting for the harvesting crew to help conduct a regular harvest, the yield was assessed on 11 February 2026 by counting the number of fruits on each tree and multiplying this number by the average fruit weight within each replicate. Pre-harvest fruit drop was estimated one week prior to the yield assessment like in trial 1. The means for each replicate were used for statistical analysis.

For both trials, a 4/5-bushel sample of fruits (approximately 70 fruits) was collected randomly from the different trees in each replicate and sent to the UF/IFAS Citrus Research and Education Center (CREC) Pilot Processing Plant (Lake Alfred, FL, USA) for juice quality analysis, including juice color, total soluble solids (TSS; °Brix), and titratable acidity (TA; %acid). A random subsample of ten fruits per replicate was evaluated for external peel color using a CR-400 chroma meter (Konica Minolta, Ramsey, NJ, USA), with measurements taken at three points around the equatorial region of each fruit. Fruit peel color was expressed as the a*/b* color ratio within the CIE L*a*b* color system, where higher positive a*/b* values correspond to a deeper red-orange color, and more negative values indicate a lighter green color. Fruit size was estimated by determining the fruit diameter of each of the 10 fruits per replicate with a digital caliper and using equation V = π d^3^/6, where d is the measured fruit diameter and V is the volume inside a sphere. Fruit size was expressed in cm^3^. The means for each replicate were used for statistical analysis.

### 4.8. Statistical Analysis

For all quantitative variables, analysis of variance was conducted using R software, version 4.5.3 (R Core Team, Vienna, Austria). Data were analyzed using linear mixed-effects models, with adjuvant treatment (without adjuvant/low pH and with adjuvant/high pH) and antibiotic treatment (none, OTC, STM, and OTC + STM) as fixed factors in a 2 × 4 factorial arrangement, and block as a random factor. When significant main effects or interactions were detected, means were separated using Tukey-adjusted comparisons (*p* < 0.05). Prior to analysis, data were evaluated for normality and homogeneity of variance. When necessary, data were log-transformed to better meet model assumptions; statistical analyses were performed on the transformed data, but non-transformed means are presented. Ordinal variables, including canopy ratings, leaf drop, and wound response variables (e.g., injection hole scores), were analyzed using non-parametric aligned rank transformation ANOVA with the ARTool package [70]. Repeated measurements were analyzed using mixed-effects models including time and its interactions with adjuvant and antibiotic treatment, followed by ANOVA to test fixed effects. Repeatedly assessed injection hole scores were analyzed using aligned rank transform ANOVA. Bark crack incidence was analyzed using a binomial generalized linear mixed model (GLMM).

## Figures and Tables

**Table 1:** Effects of adjuvant and antibiotic treatment on canopy phytotoxicity and leaf drop of ‘Valencia’ orange trees following trunk injection.

Factor	Canopy Phytotoxicity	Leaf Drop
Adjuvant		
Adjuvant (high pH)	3.5 ± 0.10 a	3.5 ± 0.10 a
No adjuvant (low pH)	1.6 ± 0.09 b	1.5 ± 0.09 b
*p*-value	<0.0001	<0.0001
Antibiotic		
None (Control)	2.4 ± 0.34 b	2.4 ± 0.34 b
OTC	2.6 ± 0.22 ab	2.6 ± 0.21 ab
STM	2.3 ± 0.30 b	2.2 ± 0.30 b
OTC + STM	2.8 ± 0.22 a	2.8 ± 0.25 a
*p*-value	0.0006	0.0079
Adjuvant × Antibiotic		
*p*-value	0.0008	0.0012

Ratings were performed 12 days after injection. Values represent mean ± standard error. Different letters within columns indicate significant differences according to Tukey’s honestly significant difference test at *p* ≤ 0.05.

**Table 2:** Effects of adjuvant and antibiotic treatment on leaf *Candidatus* Liberibacter Ct-values of ‘Valencia’ orange trees 0, 3, 6, and 9 months after injection (MAI).

Factor	Trial 1				Trial 2			
	0 MAI	3 MAI	6 MAI	9 MAI	0 MAI	3 MAI	6 MAI	9 MAI
Adjuvant								
Adjuvant (high pH)	26.1 ± 0.21	28.2 ± 0.02	27.3 ± 0.01	28.6 ± 0.38	26.8 ± 0.24	25.6 ± 0.62	25.0 ± 0.02	26.2 ± 0.01
No adjuvant (low pH)	26.1 ± 0.23	28.1 ± 0.02	27.1 ± 0.01	28.2 ± 0.40	26.5 ± 0.28	25.9 ± 0.64	24.8 ± 0.02	25.3 ± 0.01
*p*-value	0.8411	0.6411	0.8168	0.5568	0.3564	0.9417	0.9908	0.1125
Antibiotic								
None (Control)	26.2 ± 0.30	28.4 ± 0.02 ab	26.6 ± 0.02	28.5 ± 0.46	27.3 ± 0.36	25.7 ± 0.68 ab	24.5 ± 0.02 ab	25.3 ± 0.02
OTC	26.1 ± 0.30	28.8 ± 0.02 a	27.2 ± 0.02	28.1 ± 0.47	26.7 ± 0.38	26.2 ± 0.69 a	25.8 ± 0.02 a	26.6 ± 0.02
STM	26.0 ± 0.30	26.7 ± 0.02 b	26.9 ± 0.02	28.3 ± 0.47	26.5 ± 0.37	24.9 ± 0.68 b	23.9 ± 0.02 b	25.9 ± 0.02
OTC + STM	26.1 ± 0.30	28.9 ± 0.02 a	28.2 ± 0.02	28.7 ± 0.47	26.2 ± 0.36	26.0 ± 0.68 ab	25.5 ± 0.02 a	25.5 ± 0.02
*p*-value	0.9395	0.0193	0.0955	0.5813	0.1745	0.0373	0.0081	0.4036
Adjuvant × Antibiotic								
*p*-value	0.4709	0.8431	0.1872	0.0169	0.1843	0.8077	0.0771	0.5096

Values represent mean ± standard error. Different letters within columns indicate significant differences according to Tukey’s honestly significant difference (HSD) test at *p* ≤ 0.05. Letters are not shown when *p* > 0.05. The adjuvant formulation differed between trials; trial 2 used a reformulated version of the adjuvant used in trial 1.

**Table 3:** Effects of adjuvant and antibiotic treatment on injection hole score, wound width, wound length, and bark crack incidence of ‘Valencia’ orange trees three and nine months after injection.

Factor	Three Months After Injection				Nine Months After Injection			
	Injection Hole Score	Wound Width (cm)	Wound Length (cm)	Bark Crack Incidence (%)	Injection Hole Score	Wound Width (cm)	Wound Length (cm)	Bark Crack Incidence (%)
Trial 1								
Adjuvant								
Adjuvant (high pH)	2.7 ± 0.06 a	1.4 ± 0.03 a	6.1 ± 0.14 a	83 ± 0.05 a	1.9 ± 0.09	2.5 ± 0.11 a	6.8 ± 0.18 a	70 ± 0.06 a
No adjuvant (low pH)	2.4 ± 0.08 b	1.0 ± 0.03 b	1.7 ± 0.14 b	28 ± 0.06 b	1.8 ± 0.09	1.5 ± 0.11 b	2.1 ± 0.18 b	27 ± 0.06 b
*p*-value	<0.0001	<0.0001	<0.0001	<0.0001	0.2261	<0.0001	<0.0001	<0.0001
Antibiotic								
None (Control)	2.2 ± 0.12 c	1.4 ± 0.04	3.7 ± 0.15	50 ± 0.09	1.5 ± 0.11 b	1.9 ± 0.11	3.6 ± 0.19 a	31 ± 0.08 b
OTC	2.8 ± 0.07 a	1.2 ± 0.04	3.7 ± 0.15	56 ± 0.09	2.2 ± 0.11 a	1.9 ± 0.11	4.6 ± 0.19 a	56 ± 0.09 ab
STM	2.5 ± 0.10 b	1.2 ± 0.04	3.9 ± 0.15	50 ± 0.09	1.7 ± 0.12 b	2.0 ± 0.11	4.3 ± 0.19 a	44 ± 0.09 ab
OTC + STM	2.8 ± 0.08 a	1.1 ± 0.04	4.3 ± 0.15	66 ± 0.09	2.1 ± 0.13 a	2.2 ± 0.11	5.2 ± 0.19 a	63 ± 0.09 a
*p*-value	<0.0001	0.1416	0.6381	0.2409	<0.0001	0.6380	0.0494	0.0180
Adjuvant × Antibiotic								
*p*-value	<0.0001	0.0460	0.5230	0.5609	0.2797	0.2446	0.3010	0.7424
Trial 2								
Adjuvant								
Adjuvant (high pH)	3.00 ± 0.00 a	1.3 ± 0.04 a	7.4 ± 0.07 a	88 ± 0.07 a	2.6 ± 0.10 a	2.2 ± 0.05 a	8.5 ± 0.07 a	96 ± 0.04 a
No adjuvant (low pH)	2.96 ± 0.04 b	1.0 ± 0.04 b	1.8 ± 0.07 b	32 ± 0.10 b	2.0 ± 0.17 b	1.1 ± 0.05 b	2.3 ± 0.07 b	40 ± 0.10 b
*p*-value	<0.0001	<0.0001	<0.0001	<0.0001	0.0016	<0.0001	<0.0001	0.0001
Antibiotic								
None (Control)	2.9 ± 0.10	1.2 ± 0.04	3.5 ± 0.10 b	40 ± 0.16	1.9 ± 0.23 ab	1.4 ± 0.06 b	3.6 ± 0.10 c	50 ± 0.17 b
OTC	3.0 ± 0.00	1.1 ± 0.04	4.8 ± 0.10 ab	75 ± 0.11	2.6 ± 0.16 a	1.7 ± 0.06 ab	5.8 ± 0.10 b	75 ± 0.11 ab
STM	3.0 ± 0.00	1.1 ± 0.04	3.5 ± 0.10 b	46 ± 0.14	2.0 ± 0.23 b	1.4 ± 0.06 b	4.1 ± 0.10 c	54 ± 0.14 b
OTC + STM	3.0 ± 0.00	1.2 ± 0.04	6.6 ± 0.10 a	73 ± 0.14	2.5 ± 0.21 ab	2.0 ± 0.06 a	8.2 ± 0.10 a	91 ± 0.09 a
*p*-value	0.2162	0.7766	<0.0001	0.0577	0.0184	0.0014	<0.0001	0.0019
Adjuvant × Antibiotic								
*p*-value	<0.0001	0.8748	0.5015	0.2540	0.2824	0.3922	0.6410	0.0249

Values represent mean ± standard error. Different letters within columns indicate significant differences according to Tukey’s honestly significant difference (HSD) test at *p* ≤ 0.05. Letters are not shown when *p* > 0.05. The adjuvant formulation differed between trials; trial 2 used a reformulated version of the adjuvant used in trial 1.

**Table 4:** Effects of adjuvant and antibiotic treatment on fruit drop, yield, and fruit quality of ‘Valencia’ orange trees.

Factor	Fruit Drop (%)	Yield (kg/Tree)	Fruit Weight (g)	Fruit Size (cm^3^)	Peel Color (a*/b* Color)
Trial 1					
Adjuvant					
Adjuvant (high pH)	6.6 ± 0.07	26.2 ± 1.39	130 ± 1.93	139 ± 3.11	−5.9 ± 0.33
No adjuvant (low pH)	6.1 ± 0.08	25.8 ± 1.49	127 ± 2.05	139 ± 3.32	−5.8 ± 0.37
*p*-value	0.2734	0.9812	0.5685	0.7675	0.7796
Antibiotic					
None (Control)	7.4 ± 0.09 a	21.2 ± 1.85 c	119 ± 2.49 b	124 ± 4.08 b	−7.1 ± 0.49 b
OTC	5.8 ± 0.10 ab	28.9 ± 1.90 ab	140 ± 2.55 a	153 ± 4.19 a	−4.8 ± 0.51 a
STM	6.9 ± 0.09 ab	23.5 ± 1.86 bc	120 ± 2.50 b	127 ± 4.11 b	−6.8 ± 0.49 b
OTC + STM	5.4 ± 0.09 b	30.5 ± 1.85 a	135 ± 2.49 a	152 ± 4.08 a	−4.7 ± 0.49 a
*p*-value	0.0125	0.0006	<0.0001	<0.0001	0.0004
Adjuvant × Antibiotic					
*p*-value	0.1934	0.7621	0.1264	0.8184	0.7685
Trial 2					
Adjuvant					
Adjuvant (high pH)	15.5 ± 0.94	18.8 ± 0.06	124 ± 0.03	129 ± 5.16	1.0 ± 0.25
No adjuvant (low pH)	14.7 ± 1.06	18.3 ± 0.07	119 ± 0.03	122 ± 5.42	1.1 ± 0.27
*p*-value	0.4129	0.9802	0.1619	0.2344	0.6787
Antibiotic					
None (Control)	17.3 ± 1.29 a	16.5 ± 0.08 b	118 ± 0.04	127 ± 5.97	0.7 ± 0.31
OTC	12.9 ± 1.36 b	21.3 ± 0.08 a	128 ± 0.04	126 ± 6.15	1.3 ± 0.32
STM	17.2 ± 1.32 ab	17.2 ± 0.08 b	117 ± 0.04	123 ± 6.04	0.8 ± 0.31
OTC + STM	13.4 ± 1.29 ab	19.3 ± 0.08 ab	124 ± 0.04	127 ± 5.97	1.2 ± 0.31
*p*-value	0.0119	0.0096	0.0616	0.9643	0.3705
Adjuvant × Antibiotic					
*p*-value	0.6745	0.8575	0.7711	0.0395	0.8397

Values represent mean ± standard error. Different letters within columns indicate significant differences according to Tukey’s honestly significant difference (HSD) test at *p* ≤ 0.05. Letters are not shown when *p* > 0.05. The adjuvant formulation differed between trials; trial 2 used a reformulated version of the adjuvant used in trial 1.

**Table 5:** Effects of adjuvant and antibiotic treatment on juice quality variables of ‘Valencia’ orange trees.

Factor	TSS (°Brix)	TA (%)	TSS/TA	Lbs. Solids/Box	Juice Color
Trial 1					
Adjuvant					
Adjuvant (high pH)	9.4 ± 0.07	0.61 ± 0.01	15.6 ± 0.02	4.9 ± 0.04	36.3 ± 0.06
No adjuvant (low pH)	9.4 ± 0.07	0.59 ± 0.01	16.1 ± 0.02	4.9 ± 0.04	36.3 ± 0.07
*p*-value	0.7526	0.2048	0.2517	0.8789	0.6853
Antibiotic					
None (Control)	8.9 ± 0.10 b	0.61 ± 0.01	14.6 ± 0.03 b	4.6 ± 0.06 b	36.1 ± 0.09 b
OTC	9.8 ± 0.10 a	0.59 ± 0.01	16.9 ± 0.03 a	5.1 ± 0.06 a	36.5 ± 0.10 a
STM	8.9 ± 0.10 b	0.62 ± 0.01	14.6 ± 0.03 b	4.7 ± 0.06 b	36.3 ± 0.10 ab
OTC + STM	9.8 ± 0.10 a	0.58 ± 0.01	17.1 ± 0.03 a	5.1 ± 0.06 a	36.4 ± 0.09 ab
*p*-value	<0.0001	0.1412	<0.0001	<0.0001	0.0315
Adjuvant × Antibiotic					
*p*-value	0.3378	0.5190	0.4682	0.1707	0.2660
Trial 2					
Adjuvant					
Adjuvant (high pH)	9.7 ± 0.09 b	0.98 ± 0.03 b	10.0 ± 0.24 a	4.6 ± 0.06 b	35.7 ± 0.09
No adjuvant (low pH)	9.9 ± 0.10 a	1.03 ± 0.03 a	9.7 ± 0.25 b	4.8 ± 0.07 a	35.5 ± 0.10
*p*-value	0.0131	0.0008	0.0196	0.0065	0.5244
Antibiotic					
None (Control)	9.4 ± 0.12 b	1.04 ± 0.03 a	9.1 ± 0.27 b	4.4 ± 0.08 b	35.3 ± 0.11 b
OTC	10.2 ± 0.13 a	0.99 ± 0.03 ab	10.4 ± 0.28 a	4.9 ± 0.09 a	36.0 ± 0.12 a
STM	9.3 ± 0.13 b	1.01 ± 0.03 ab	9.3 ± 0.27 b	4.4 ± 0.08 b	35.4 ± 0.12 b
OTC + STM	10.1 ± 0.12 a	0.96 ± 0.03 b	10.6 ± 0.27 a	4.8 ± 0.08 a	35.8 ± 0.11 a
*p*-value	<0.0001	0.0422	<0.0001	<0.0001	<0.0001
Adjuvant × Antibiotic					
*p*-value	0.3856	0.9082	0.8752	0.4358	0.4766

Values represent mean ± standard error. Different letters within columns indicate significant differences according to Tukey’s honestly significant difference (HSD) test at *p* ≤ 0.05. Letters are not shown when *p* > 0.05. The adjuvant formulation differed between trials; trial 2 used a reformulated version of the adjuvant used in trial 1.

## Data Availability

The raw data supporting the conclusions of this article will be made available by the authors on request.

## Supplementary Materials

The following supporting information can be downloaded at https://www.mdpi.com/article/10.3390/antibiotics15090892/s1, Tables S1–S10: Supporting information on tree growth, canopy health, phytotoxicity, *C*Las detection, wound response, fruit drop, yield, and fruit quality.
