# Supplementary material for: Physiological and Horticultural Responses of HLB-Affected ‘Valencia’ Orange Trees to Trunk-Injected Oxytetracycline and Streptomycin Formulations Differing in Adjuvant and pH
Source: Antibiotics (Basel). 2026 Sep 11;15(9):892. doi: 10.3390/antibiotics15090892 (PMC13603316; doi:10.3390/antibiotics15090892)
Supplement: Supplementary file 1 [file antibiotics-15-00892-s001.zip › antibiotics-4538938-supplementary.pdf]

Supplementary Materials

TITLE: Physiological and Horticultural Responses of HLB-Affected 'Valencia' Orange Trees to Trunk-Injected Oxytetracycline and Streptomycin Formulations Differing in Adjuvant and pH

Contents

Table S1. Effects of adjuvant and antibiotic treatments and their interaction on rootstock diameter, scion diameter, and canopy volume increments of 'Valencia' orange trees from 0 to 12 months after injection in trial 1. 2

Table S2. Effects of adjuvant and antibiotic treatments and their interaction on rootstock diameter, scion diameter, and canopy volume increments of 'Valencia' orange trees from 0 to 12 months after injection in trial 2. 3

Table S3. Effects of adjuvant and antibiotic treatments and their interaction on HLB severity and canopy density ratings of 'Valencia' orange trees at the initial and final assessments in trial 1. 4

Table S4. Effects of adjuvant and antibiotic treatments and their interaction on HLB severity and canopy density ratings of 'Valencia' orange trees at the initial and final assessments in trial 2. 5

Table S5. Adjuvant × antibiotic interaction effects on canopy phytotoxicity and leaf drop ratings of 'Valencia' orange trees 12 days after injection in trial 1. 6

Table S6. Adjuvant × antibiotic interaction effects on leaf *Candidatus* Liberibacter asiaticus Ct-values of 'Valencia' orange trees 0, 3, 6, and 9 months after injection (MAI) in trials 1 and 2. 7

Table S7. Repeated measures ANOVA for adjuvant, antibiotic, and time effects on leaf *Candidatus* Liberibacter asiaticus Ct-values of 'Valencia' orange trees in trials 1 and 2. 8

Table S8. Adjuvant × antibiotic interaction effects on injection-hole score, wound width, wound length, and bark crack incidence of 'Valencia' orange trees three and nine months after injection in trials 1 and 2. 9

Table S9. Repeated-measures analyses of adjuvant, antibiotic, and time effects on wound responses of 'Valencia' orange trees in trials 1 and 2 following injection. 10

Table S10. Adjuvant × antibiotic interaction effects on fruit drop, yield, and fruit quality of 'Valencia' orange trees in trials 1 and 2. 11

Table S1. Effects of adjuvant and antibiotic treatments and their interaction on rootstock diameter, scion diameter, and canopy volume increments of 'Valencia' orange trees from 0 to 12 months after injection in trial 1.

**Factor**	**Rootstock diameter increment (cm)**	**Scion diameter increment (cm)**	**Canopy volume increment (m^3)**
Adjuvant			
Adjuvant (high pH)	0.1 ± 0.03	0.3 ± 0.11	1.2 ± 0.56
No adjuvant (low pH)	0.1 ± 0.03	0.1 ± 0.11	1.0 ± 0.56
p-value	0.3854	0.3607	0.1540
Antibiotic			
None (Control)	0.1 ± 0.05	-0.0 ± 0.15	1.3 ± 0.57
OTC	0.1 ± 0.05	0.4 ± 0.15	0.8 ± 0.57
STM	0.2 ± 0.05	0.1 ± 0.15	1.1 ± 0.57
OTC + STM	0.1 ± 0.05	0.2 ± 0.15	1.1 ± 0.57
p-value	0.6234	0.2028	0.1342
Adjuvant × Antibiotic			
Adjuvant (high pH) + None (Control)	0.1 ± 0.06	-0.0 ± 0.22	1.2 ± 0.59
Adjuvant (high pH) + OTC	0.1 ± 0.06	0.8 ± 0.22	1.0 ± 0.59
Adjuvant (high pH) + STM	0.2 ± 0.06	0.2 ± 0.22	1.2 ± 0.59
Adjuvant (high pH) + OTC + STM	0.1 ± 0.06	0.1 ± 0.22	1.3 ± 0.59
No adjuvant (low pH) + None (Control)	0.1 ± 0.06	0.0 ± 0.22	1.4 ± 0.59
No adjuvant (low pH) + OTC	0.1 ± 0.06	0.1 ± 0.22	0.6 ± 0.59
No adjuvant (low pH) + STM	0.1 ± 0.06	0.1 ± 0.22	1.0 ± 0.59
No adjuvant (low pH) + OTC + STM	0.1 ± 0.06	0.2 ± 0.22	0.9 ± 0.59
p-value	0.8535	0.3260	0.4856

Values represent mean ± standard error. No significant main effects or adjuvant × antibiotic interactions were detected ($p > 0.05$).

Table S2. Effects of adjuvant and antibiotic treatments and their interaction on rootstock diameter, scion diameter, and canopy volume increments of 'Valencia' orange trees from 0 to 12 months after injection in trial 2.

**Factor**	**Rootstock diameter increment (cm)**	**Scion diameter increment (cm)**	**Canopy volume increment (m^3)**
Adjuvant			
Adjuvant (high pH)	0.8 ± 0.29	0.2 ± 0.44	-0.8 ± 0.28
No adjuvant (low pH)	0.8 ± 0.29	-0.1 ± 0.44	-0.5 ± 0.28
p-value	0.9266	0.7484	0.2583
Antibiotic			
None (Control)	0.6 ± 0.43	0.2 ± 0.58	-0.5 ± 0.36
OTC	0.5 ± 0.37	-0.7 ± 0.57	-0.9 ± 0.35
STM	1.1 ± 0.38	0.1 ± 0.61	-0.7 ± 0.34
OTC + STM	1.0 ± 0.43	0.6 ± 0.61	-0.6 ± 0.35
p-value	0.6386	0.4593	0.8528
Adjuvant × Antibiotic			
Adjuvant (high pH) + None (Control)	0.7 ± 0.66	0.1 ± 0.79	-0.4 ± 0.46
Adjuvant (high pH) + OTC	0.4 ± 0.49	0.1 ± 0.79	-1.0 ± 0.46
Adjuvant (high pH) + STM	1.0 ± 0.52	-0.3 ± 0.84	-0.7 ± 0.46
Adjuvant (high pH) + OTC + STM	1.2 ± 0.60	0.8 ± 0.84	-1.2 ± 0.46
No adjuvant (low pH) + None (Control)	0.6 ± 0.55	0.2 ± 0.79	-0.5 ± 0.48
No adjuvant (low pH) + OTC	0.6 ± 0.55	-1.5 ± 0.77	-0.8 ± 0.46
No adjuvant (low pH) + STM	1.3 ± 0.55	0.6 ± 0.84	-0.7 ± 0.44
No adjuvant (low pH) + OTC + STM	0.7 ± 0.60	0.4 ± 0.84	-0.0 ± 0.46
p-value	0.8902	0.4726	0.4723

Values represent mean ± standard error. No significant main effects or adjuvant × antibiotic interactions were detected ($p > 0.05$).

Table S3. Effects of adjuvant and antibiotic treatments and their interaction on HLB severity and canopy density ratings of 'Valencia' orange trees at the initial and final assessments in trial 1.

Factor	Initial assessment		Final assessment	
	**HLB severity**	**Canopy density**	**HLB severity**	**Canopy density**
Adjuvant				
Adjuvant (high pH)	3.2 ± 0.09	4.0 ± 0.07	2.5 ± 0.05	4.0 ± 0.08
No adjuvant (low pH)	3.2 ± 0.07	3.9 ± 0.08	2.6 ± 0.06	3.9 ± 0.08
p-value	0.9060	0.2654	0.2128	0.4886
Antibiotic				
None (Control)	3.2 ± 0.11	3.8 ± 0.10	2.6 ± 0.08	3.8 ± 0.11
OTC	3.3 ± 0.11	3.9 ± 0.10	2.5 ± 0.08	4.0 ± 0.11
STM	3.2 ± 0.12	4.0 ± 0.10	2.6 ± 0.09	3.8 ± 0.11
OTC + STM	3.2 ± 0.11	3.9 ± 0.10	2.6 ± 0.08	4.0 ± 0.10
p-value	0.9571	0.2724	0.6608	0.3196
Adjuvant × Antibiotic				
Adjuvant (high pH) + None (Control)	3.3 ± 0.16	3.8 ± 0.12	2.5 ± 0.11	3.8 ± 0.14
Adjuvant (high pH) + OTC	3.2 ± 0.14	4.2 ± 0.14	2.4 ± 0.12	4.2 ± 0.14
Adjuvant (high pH) + STM	3.2 ± 0.20	4.0 ± 0.11	2.7 ± 0.10	3.8 ± 0.16
Adjuvant (high pH) + OTC + STM	3.2 ± 0.19	4.0 ± 0.15	2.5 ± 0.11	4.1 ± 0.15
No adjuvant (low pH) + None (Control)	3.2 ± 0.16	3.8 ± 0.16	2.7 ± 0.12	3.9 ± 0.18
No adjuvant (low pH) + OTC	3.3 ± 0.16	3.7 ± 0.14	2.6 ± 0.10	3.9 ± 0.16
No adjuvant (low pH) + STM	3.2 ± 0.13	4.1 ± 0.16	2.6 ± 0.14	3.8 ± 0.15
No adjuvant (low pH) + OTC + STM	3.3 ± 0.11	3.8 ± 0.14	2.8 ± 0.13	3.9 ± 0.14
p-value	0.8882	0.1964	0.1766	0.4527

Values represent mean ± standard error. No significant main effects or adjuvant × antibiotic interactions were detected for HLB severity or canopy density at either assessment ($p > 0.05$).

Table S4. Effects of adjuvant and antibiotic treatments and their interaction on HLB severity and canopy density ratings of 'Valencia' orange trees at the initial and final assessments in trial 2.

Factor	Initial assessment		Final assessment	
	**HLB severity**	**Canopy density**	**HLB severity**	**Canopy density**
Adjuvant				
Adjuvant (high pH)	2.6 ± 0.10	3.6 ± 0.11	2.8 ± 0.14	3.6 ± 0.13
No adjuvant (low pH)	2.7 ± 0.11	3.8 ± 0.10	3.1 ± 0.20	3.3 ± 0.19
p-value	0.2588	0.1629	0.4126	0.4016
Antibiotic				
None (Control)	2.8 ± 0.20	3.5 ± 0.20	3.2 ± 0.22	3.5 ± 0.28
OTC	2.5 ± 0.12	3.9 ± 0.14	2.6 ± 0.24	3.5 ± 0.22
STM	2.6 ± 0.15	3.7 ± 0.13	2.9 ± 0.19	3.7 ± 0.20
OTC + STM	2.8 ± 0.12	3.6 ± 0.14	3.2 ± 0.29	3.0 ± 0.24
p-value	0.3470	0.2710	0.4867	0.1839
Adjuvant × Antibiotic				
Adjuvant (high pH) + None (Control)	2.6 ± 0.24	3.2 ± 0.25	3.2 ± 0.25	3.4 ± 0.43
Adjuvant (high pH) + OTC	2.4 ± 0.15	3.8 ± 0.20	2.2 ± 0.21	3.7 ± 0.19
Adjuvant (high pH) + STM	2.6 ± 0.20	3.7 ± 0.17	2.9 ± 0.20	3.8 ± 0.25
Adjuvant (high pH) + OTC + STM	2.8 ± 0.25	3.7 ± 0.20	3.3 ± 0.20	3.3 ± 0.20
No adjuvant (low pH) + None (Control)	3.0 ± 0.32	3.8 ± 0.25	3.1 ± 0.40	3.5 ± 0.42
No adjuvant (low pH) + OTC	2.6 ± 0.21	3.9 ± 0.20	3.1 ± 0.43	3.2 ± 0.43
No adjuvant (low pH) + STM	2.6 ± 0.23	3.8 ± 0.21	2.9 ± 0.32	3.5 ± 0.31
No adjuvant (low pH) + OTC + STM	2.8 ± 0.11	3.6 ± 0.20	3.1 ± 0.52	2.8 ± 0.40
p-value	0.6973	0.4716	0.3106	0.9727

Values represent mean ± standard error. No significant main effects or adjuvant × antibiotic interactions were detected for HLB severity or canopy density at either assessment ($p > 0.05$).

Table S5. Adjuvant × antibiotic interaction effects on canopy phytotoxicity and leaf drop ratings of 'Valencia' orange trees 12 days after injection in trial 1.

Factor	Canopy phytotoxicity	Leaf drop
Adjuvant × Antibiotic		
Adjuvant (high pH) + None (Control)	3.7 ± 0.18 a	3.7 ± 0.15 a
Adjuvant (high pH) + OTC	3.3 ± 0.18 ab	3.3 ± 0.18 ab
Adjuvant (high pH) + STM	3.3 ± 0.28 ab	3.2 ± 0.29 ab
Adjuvant (high pH) + OTC + STM	3.6 ± 0.11 ab	3.6 ± 0.11 ab
No adjuvant (low pH) + None (Control)	1.1 ± 0.07 b	1.1 ± 0.07 b
No adjuvant (low pH) + OTC	1.9 ± 0.14 ab	1.9 ± 0.14 ab
No adjuvant (low pH) + STM	1.2 ± 0.12 ab	1.2 ± 0.12 ab
No adjuvant (low pH) + OTC + STM	2.0 ± 0.12 ab	1.9 ± 0.17 ab
p-value	0.0008	0.0012

Values represent mean ± standard error. Different letters within columns indicate significant differences according to Tukey's honestly significant difference test at $p \leq 0.05$.

Table S6. Adjuvant × antibiotic interaction effects on leaf *Candidatus* Liberibacter asiaticus Ct-values of 'Valencia' orange trees 0, 3, 6, and 9 months after injection (MAI) in trials 1 and 2.

Factor	0 MAI	3 MAI	6 MAI	9 MAI
	**Trial 1**			
Adjuvant × Antibiotic				
Adjuvant (high pH) + None (Control)	26.4 ± 0.41	28.2 ± 0.03	26.8 ± 0.02	28.6 ± 0.58 a
Adjuvant (high pH) + OTC	25.9 ± 0.37	28.9 ± 0.03	26.6 ± 0.02	28.9 ± 0.54 a
Adjuvant (high pH) + STM	26.3 ± 0.39	26.6 ± 0.03	27.0 ± 0.02	27.7 ± 0.58 a
Adjuvant (high pH) + OTC + STM	26.0 ± 0.41	29.3 ± 0.03	28.8 ± 0.02	29.0 ± 0.58 a
No adjuvant (low pH) + None (Control)	26.0 ± 0.41	28.5 ± 0.03	26.5 ± 0.02	28.4 ± 0.58 a
No adjuvant (low pH) + OTC	26.4 ± 0.47	28.7 ± 0.03	28.1 ± 0.03	26.9 ± 0.65 a
No adjuvant (low pH) + STM	25.7 ± 0.43	26.9 ± 0.03	26.7 ± 0.02	29.0 ± 0.61 a
No adjuvant (low pH) + OTC + STM	26.3 ± 0.41	28.4 ± 0.03	27.5 ± 0.02	28.3 ± 0.61 a
p-value	0.4709	0.8431	0.1872	0.0169
	**Trial 2**			
Adjuvant (high pH) + None (Control)	27.3 ± 0.51	25.7 ± 0.77	23.5 ± 0.03	26.0 ± 0.03
Adjuvant (high pH) + OTC	27.3 ± 0.44	25.8 ± 0.73	25.9 ± 0.03	26.9 ± 0.02
Adjuvant (high pH) + STM	26.3 ± 0.46	24.9 ± 0.74	24.6 ± 0.03	26.5 ± 0.02
Adjuvant (high pH) + OTC + STM	26.3 ± 0.51	25.9 ± 0.77	25.8 ± 0.03	25.2 ± 0.03
No adjuvant (low pH) + None (Control)	27.3 ± 0.51	25.7 ± 0.77	25.4 ± 0.03	24.6 ± 0.03
No adjuvant (low pH) + OTC	25.6 ± 0.63	27.2 ± 0.86	25.5 ± 0.03	25.9 ± 0.03
No adjuvant (low pH) + STM	26.8 ± 0.58	24.8 ± 0.82	22.8 ± 0.03	25.0 ± 0.03
No adjuvant (low pH) + OTC + STM	26.2 ± 0.51	26.2 ± 0.77	25.2 ± 0.03	25.7 ± 0.03
p-value	0.1843	0.8077	0.0771	0.5096

Values represent mean ± standard error. Different letters within columns indicate significant differences according to Tukey's honestly significant difference (HSD) test at $p \leq 0.05$. Letters are not shown when $p > 0.05$.

Table S7. Repeated measures ANOVA for adjuvant, antibiotic, and time effects on leaf *Candidatus* Liberibacter asiaticus Ct-values of 'Valencia' orange trees in trials 1 and 2.

**Factor**	***p-value***
**Trial 1**	
Adjuvant	0.4293
Antibiotic	0.0216
Time	<0.0001
Adjuvant × Antibiotic	0.5871
Adjuvant × Time	0.9550
Antibiotic × Time	0.1262
Adjuvant × Antibiotic × Time	0.1876
**Trial 2**	
Adjuvant	0.2396
Antibiotic	0.0348
Time	<0.0001
Adjuvant × Antibiotic	0.5002
Adjuvant × Time	0.3720
Antibiotic × Time	0.1315
Adjuvant × Antibiotic × Time	0.1911

Candidatus Liberibacter asiaticus Ct-values were analyzed using linear mixed-effects models.

Table S8. Adjuvant × antibiotic interaction effects on injection-hole score, wound width, wound length, and bark crack incidence of 'Valencia' orange trees three and nine months after injection in trials 1 and 2.

Factor	Three months after injection				Nine months after injection			
	Injection-hole score	Wound width (cm)	Wound length (cm)	Bark crack incidence (%)	Injection-hole score	Wound width (cm)	Wound length (cm)	Bark crack incidence (%)
**Trial 1**								
Adjuvant × Antibiotic								
Adjuvant (high pH) + None (Control)	2.6 ± 0.13 a	1.9 ± 0.05 a	5.3 ± 0.17	75 ± 0.11	1.6 ± 0.18	2.4 ± 0.12	5.7 ± 0.21	50 ± 0.13
Adjuvant (high pH) + OTC	2.8 ± 0.10 b	1.4 ± 0.05 ab	5.5 ± 0.17	88 ± 0.09	2.1 ± 0.18	2.2 ± 0.12	6.2 ± 0.21	81 ± 0.10
Adjuvant (high pH) + STM	2.7 ± 0.12 ab	1.4 ± 0.05 ab	6.5 ± 0.17	81 ± 0.10	1.8 ± 0.17	2.5 ± 0.12	7.1 ± 0.21	69 ± 0.12
Adjuvant (high pH) + OTC + STM	2.8 ± 0.10 ab	1.2 ± 0.05 bc	7.1 ± 0.17	88 ± 0.09	2.2 ± 0.17	2.8 ± 0.12	8.1 ± 0.21	81 ± 0.10
No adjuvant (low pH) + None (Control)	1.7 ± 0.12 b	1.0 ± 0.05 c	2.1 ± 0.17	25 ± 0.11	1.3 ± 0.12	1.5 ± 0.12	1.4 ± 0.21	12 ± 0.09
No adjuvant (low pH) + OTC	2.9 ± 0.09 ab	1.0 ± 0.05 c	1.9 ± 0.17	25 ± 0.11	2.3 ± 0.12	1.6 ± 0.12	2.9 ± 0.21	31 ± 0.12
No adjuvant (low pH) + STM	2.2 ± 0.14 ab	1.0 ± 0.05 c	1.2 ± 0.17	19 ± 0.10	1.6 ± 0.18	1.5 ± 0.12	1.6 ± 0.21	19 ± 0.10
No adjuvant (low pH) + OTC + STM	2.7 ± 0.12 a	1.1 ± 0.05 bc	1.6 ± 0.17	44 ± 0.13	2.0 ± 0.18	1.6 ± 0.12	2.3 ± 0.21	44 ± 0.13
p-value	<0.0001	0.0460	0.5230	0.5609	0.2797	0.2446	0.3010	0.7424
**Trial 2**								
Adjuvant × Antibiotic								
Adjuvant (high pH) + None (Control)	3.00 ± 0.00 a	1.4 ± 0.05	5.8 ± 0.14	80 ± 0.20	2.4 ± 0.24	1.9 ± 0.08	5.8 ± 0.14	80 ± 0.20 a
Adjuvant (high pH) + OTC	3.00 ± 0.00 a	1.2 ± 0.05	7.6 ± 0.14	89 ± 0.11	2.7 ± 0.17	2.3 ± 0.08	9.3 ± 0.14	100 ± 0.00 a
Adjuvant (high pH) + STM	3.00 ± 0.00 a	1.2 ± 0.05	5.8 ± 0.14	83 ± 0.17	2.5 ± 0.22	1.8 ± 0.08	6.7 ± 0.14	100 ± 0.00 a
Adjuvant (high pH) + OTC + STM	3.00 ± 0.00 a	1.3 ± 0.05	10.3 ± 0.14	100 ± 0.00	2.6 ± 0.24	2.8 ± 0.08	12.3 ± 0.14	100 ± 0.00 a
No adjuvant (low pH) + None (Control)	2.80 ± 0.20 a	1.0 ± 0.05	1.3 ± 0.14	0 ± 0.00	1.4 ± 0.24	1.0 ± 0.08	1.4 ± 0.14	20 ± 0.20 a
No adjuvant (low pH) + OTC	3.00 ± 0.00 a	1.0 ± 0.05	2.1 ± 0.14	57 ± 0.20	2.4 ± 0.30	1.2 ± 0.08	2.4 ± 0.14	43 ± 0.20 a
No adjuvant (low pH) + STM	3.00 ± 0.00 a	1.0 ± 0.05	1.1 ± 0.14	14 ± 0.14	1.6 ± 0.30	1.1 ± 0.08	1.4 ± 0.14	14 ± 0.14 a
No adjuvant (low pH) + OTC + STM	3.00 ± 0.00 a	1.0 ± 0.05	2.8 ± 0.14	50 ± 0.22	2.3 ± 0.33	1.2 ± 0.08	4.0 ± 0.14	83 ± 0.17 a
p-value	<0.0001	0.8748	0.5015	0.2540	0.2824	0.3922	0.6410	0.0249

Values represent mean ± standard error. Different letters within columns indicate significant differences according to Tukey's honestly significant difference (HSD) test at $p \leq 0.05$. Letters are not shown when $p > 0.05$.

Table S9. Repeated-measures analyses of adjuvant, antibiotic, and time effects on wound responses of 'Valencia' orange trees in trials 1 and 2 following injection.

**Factor**	**p-value**			
	**Injection hole score**	**Wound width**	**Wound length**	**Bark crack incidence**
	**Trial 1**			
Adjuvant	0.0004	<0.0001	<0.0001	0.0008
Antibiotic	<0.0001	0.6144	0.7361	0.8718
Time	<0.0001	<0.0001	<0.0001	0.0133
Adjuvant × Antibiotic	0.0001	0.4600	0.5927	0.3849
Adjuvant × Time	0.0222	0.0017	0.1882	0.5908
Antibiotic × Time	0.3486	0.1213	0.0171	0.4988
Adjuvant × Antibiotic × Time	0.6992	0.1486	0.2552	0.8998
	**Trial 2**			
Adjuvant	<0.0001	<0.0001	<0.0001	<0.0001
Antibiotic	<0.0001	0.0141	<0.0001	0.9792
Time	<0.0001	<0.0001	<0.0001	0.6188
Adjuvant × Antibiotic	0.0198	0.1939	0.1299	0.5297
Adjuvant × Time	<0.0001	<0.0001	0.0307	0.4831
Antibiotic × Time	<0.0001	0.0013	0.0050	0.9073
Adjuvant × Antibiotic × Time	0.0017	0.0472	0.3775	0.9251

Wound length and wound width were analyzed using linear mixed models; injection-hole score was analyzed using aligned rank transform mixed models; and bark crack incidence was analyzed using binomial generalized linear mixed models.

Table S10. Adjuvant × antibiotic interaction effects on fruit drop, yield, and fruit quality of 'Valencia' orange trees in trials 1 and 2.

Factor	Fruit drop (%)	Yield (kg/tree)	Fruit weight (g)	Fruit size (cm^3)	Peel color (a*/b* color)
**Trial 1**					
Adjuvant × Antibiotic					
Adjuvant (high pH) + None (Control)	7.0 ± 0.12	21.1 ± 2.50	116 ± 3.29	124 ± 5.46	-7.5 ± 0.69
Adjuvant (high pH) + OTC	6.5 ± 0.11	29.5 ± 2.28	143 ± 3.01	151 ± 4.99	-4.9 ± 0.62
Adjuvant (high pH) + STM	7.0 ± 0.12	24.5 ± 2.38	120 ± 3.14	126 ± 5.21	-6.5 ± 0.65
Adjuvant (high pH) + OTC + STM	6.0 ± 0.12	29.2 ± 2.50	137 ± 3.29	154 ± 5.46	-4.9 ± 0.69
No adjuvant (low pH) + None (Control)	7.7 ± 0.12	21.3 ± 2.50	122 ± 3.29	124 ± 5.46	-6.8 ± 0.69
No adjuvant (low pH) + OTC	4.6 ± 0.14	28.0 ± 2.88	134 ± 3.77	157 ± 6.28	-4.6 ± 0.81
No adjuvant (low pH) + STM	6.9 ± 0.13	22.3 ± 2.67	120 ± 3.50	128 ± 5.83	-7.2 ± 0.74
No adjuvant (low pH) + OTC + STM	4.8 ± 0.12	31.9 ± 2.50	134 ± 3.29	150 ± 5.46	-4.5 ± 0.69
p-value	0.1934	0.7621	0.1264	0.8184	0.7685
**Trial 2**					
Adjuvant × Antibiotic					
Adjuvant (high pH) + None (Control)	17.8 ± 1.77	16.2 ± 0.10	122 ± 0.04	135 ± 7.21 a	0.7 ± 0.39
Adjuvant (high pH) + OTC	13.9 ± 1.56	20.7 ± 0.09	130 ± 0.04	126 ± 6.66 a	1.3 ± 0.35
Adjuvant (high pH) + STM	17.9 ± 1.62	17.7 ± 0.09	116 ± 0.04	119 ± 6.82 a	0.7 ± 0.37
Adjuvant (high pH) + OTC + STM	12.7 ± 1.77	20.2 ± 0.10	129 ± 0.04	136 ± 7.21 a	1.1 ± 0.39
No adjuvant (low pH) + None (Control)	16.9 ± 1.77	16.8 ± 0.10	115 ± 0.04	120 ± 7.21 a	0.7 ± 0.39
No adjuvant (low pH) + OTC	10.8 ± 2.17	22.4 ± 0.12	125 ± 0.05	124 ± 8.40 a	1.2 ± 0.47
No adjuvant (low pH) + STM	15.9 ± 2.01	16.6 ± 0.11	119 ± 0.05	130 ± 7.91 a	1.1 ± 0.44
No adjuvant (low pH) + OTC + STM	14.1 ± 1.77	18.4 ± 0.10	120 ± 0.04	118 ± 7.21 a	1.3 ± 0.39
p-value	0.6745	0.8575	0.7711	0.0395	0.8397

Values represent mean ± standard error. Different letters within columns indicate significant differences according to Tukey's honestly significant difference (HSD) test at $p \leq 0.05$. Letters are not shown when $p > 0.05$.
